# Biomass-derived Graphite-like Carbon as a Sustainable Cathode Material for Lithium Oxygen Batteries

**DOI:** 10.3390/ma19214632

**Published:** 2026-10-31

**Authors:** Da-Jung Kang, Cheol-Hui Ryu, Gwang-Hee Lee, Kwang-Mo Seong, Jung-Eun Park

**Affiliations:** 1 Center of Bio-Resources Recycling, Institute for Advanced Engineering, Yongin 17180, Republic of Korea; 2 Center of Materials Science and Chemical Engineering, Institute for Advanced Engineering, Yongin 17180, Republic of Korea; 3 Interior Materials Development Team, Hyundai Motor Group, 150, Hyundaiyeonguso-Ro, Namyang-Eup, Manse-Gu, Hwaseong-Si 18280, Republic of Korea; paektu2000@hyundai.com

**Keywords:** spent mushroom substrate, biomass, graphite-like carbon, KOH activation, catalytic graphitization, lithium–oxygen battery, cathode

## Abstract

Lithium–oxygen batteries (LOBs) offer the highest theoretical specific energy among metal–air chemistries, but their practical deployment is limited by the sluggish reversibility of Li_2_O_2_ formation/decomposition on the cathode, which requires a porous, electronically conductive carbon combining mesoporous transport with graphitic domains. Here, we report a two-step, KOH-assisted thermal treatment route for producing graphite-like porous carbon that converts spent mushroom substrate (SMS)—a lignin-rich agro-industrial waste generated at >5 million tonnes per year in East Asia—into a graphite-like porous carbon (G-SMS) for LOB cathodes. SMS derived from Pleurotus ostreatus cultivated on an oak–sawdust substrate was carbonized at 800 °C (1 h, 3 °C min^−1^), followed by KOH-assisted heat treatment at 1200–1800 °C. A KOH/C ratio of 0.5 was selected for the detailed graphitization series for its reagent economy and carbon yield: the 1.0 ratio gave ~10 wt% lower production yield and, at 1800 °C, lower carbon content (97.6 wt%) with residual ash (1.7 wt%), for no compensating benefit. Within the KOH/C = 0.5 series, G-SMS-1800/0.5 combined the smallest interlayer spacing (d_002_ = 0.347 nm), the lowest Raman I_D/I_G (0.69), the largest mesopore fraction (96.9%) and mesopore volume (0.31 cm^3^ g^−1^), and the highest powder conductivity (8607 S m^−1^ at 2000 kgf), giving the highest mesopore-volume-weighted conductivity descriptor of the matrix, V_Meso × σ = 2668 cm^3^ g^−1^ S m^−1^. This sample was identified as the most robust host, and its cycling stability was benchmarked against Super P in a modified CR2032 LOB cell (1 M LiTFSI in N,N-dimethylacetamide, 2.0 atm O_2_): G-SMS-1800/0.5 sustained 330 cycles, whereas Super P reached only 200 cycles under identical conditions, a ~1.65-fold improvement. KOH-mediated catalytic graphitization thus lowers the thermal budget of biomass graphitization while providing a conductive, mesopore-dominated host for Li_2_O_2_ deposition, providing a potential route for the valorization of spent mushroom substrate into graphite-like porous carbon for LOB cathodes.

## 1. Introduction

The decarbonization of the transportation and grid-storage sectors is driving the search for post-lithium-ion chemistries with higher gravimetric energy densities. Among the candidates, aprotic lithium–oxygen batteries (LOBs) stand out because the 2 Li + O_2_ ⇌ Li_2_O_2_ reaction gives the highest theoretical specific energy of any metal–air couple [1,2]. However, three coupled issues still limit LOB cycling: (i) the sluggish oxygen reduction/evolution kinetics on the cathode; (ii) the insulating character of solid Li_2_O_2_, which passivates the electrode; and (iii) the parasitic decomposition of both the electrolyte and the carbon host under the superoxide-rich environment of the discharge product [3,4].

Cathode design is therefore the single most influential lever on LOB performance. An ideal cathode should offer (a) a large, mesopore-dominated void volume that accommodates the growth of insulating Li_2_O_2_ without pore clogging, (b) an electronically conductive, graphitic backbone that supports the outer-sphere electron transfer needed for a surface-mediated Li_2_O_2_ growth pathway, and (c) low cost and low embodied CO_2_ [5,6,7]. Synthetic graphites and carbon nanotubes satisfy (a) and (b) but at a high thermal and financial cost—synthetic graphite is produced by Acheson-type graphitization at 2800–3000 °C and carries an embodied CO_2_ intensity of 8–12 kg CO_2_ per kg [8,9].

Biomass-derived carbons are an attractive alternative because they are renewable and inherently porous, and their lignin fraction is a known precursor for graphitizable char [10,11,12]. Spent mushroom substrate (SMS)—the sawdust/straw block left after fruiting-body harvest—is particularly interesting: it is generated at more than five million tonnes per year in Korea, China and Japan combined, is landfilled or incinerated at a net cost to farmers, and contains 25–40 wt% lignin because the mushroom mycelium selectively consumes cellulose and hemicellulose [13,14]. Prior work has valorized SMS mostly as biochar, soil amendment or adsorbent [15,16]; its use as a battery-grade carbon precursor is still very limited.

The remaining challenge is graphitization. Non-catalytic graphitization of lignocellulosic char requires ≥2500 °C to reach a graphitic (002) spacing of 0.336 nm [17]. Catalytic graphitization with Fe, Ni or Co lowers this to 1000–1400 °C but introduces metallic impurities that must be leached with strong acids, generating a secondary waste stream [18,19,20]. Recent studies show that alkali metals—particularly potassium introduced through KOH activation—can play a dual role: K vapor intercalates the turbostratic carbon layers, restructures them into graphitic domains at ≤1500 °C, and simultaneously etches micro/mesopores, so that a single reagent controls both crystallinity and porosity [21,22,23,24].

Therefore, SMS is carbonized at 800 °C and then subjected to a KOH-co-treated heat-treatment in the range 1200–1800 °C. We correlate the KOH/C mass ratio and the graphitization temperature with the crystallographic order (XRD, Raman), the texture (N_2_ physisorption), the powder electrical conductivity, and the cycling stability of the resulting carbons against a commercial Super P reference electrode.

## 2. Experimental Section

### 2.1. Materials

SMS derived from Pleurotus ostreatus cultivation on an oak-sawdust substrate was supplied by a local farm (Yongin, Republic of Korea) (Table S1). Potassium hydroxide (≥85%, Sigma-Aldrich, St. Louis, MO, USA), lithium bis(trifluoromethanesulfonyl)imide (LiTFSI, ≥98%, Alfa Aesar, Ward Hill, MA, USA), and N,N-dimethylacetamide (DMAc, anhydrous, 99.8%, Sigma-Aldrich, St. Louis, MO, USA) were used as received. Super P carbon black (MTI Korea, Seoul, Republic of Korea) was employed as a reference cathode carbon.

### 2.2. Carbonization and KOH-Assisted Graphitization

SMS was air-dried, ground and screened (<250 µm), and then dried at 105 °C for 24 h. A screening matrix (500–1000 °C; 1, 3, 5 h; 1.5–10 °C min^−1^ under N_2_; Supporting Information Tables S2 and S3) identified 800 °C, 1 h and 3 °C min^−1^ as the optimum carbonization condition, giving the highest fixed-carbon yield (80.1 wt%) and the lowest ash-normalized oxygen content. This char is hereafter denoted C-SMS-800.

For the second, graphitization step, C-SMS-800 was ground with solid KOH in a KOH/C mass ratio of 0.5 and 1.0 in an agate mortar. The mixture was loaded into a graphite crucible and heated in a tube furnace under flowing N_2_ (200 mL min^−1^) at 10 °C min^−1^ to a target temperature of 1200, 1400, 1600 or 1800 °C, held for 1 h, and cooled naturally. The product was washed with 1 M HCl (60 °C, 2 h) and then with deionized water until the filtrate reached pH 6.5, and freeze-dried. Samples are labeled G-SMS-T/x where T is the graphitization temperature (°C) and x is the KOH/C mass ratio. Both KOH/C = 0.5 and 1.0 series were evaluated in terms of production yield, elemental composition, and textural properties (Table 1 and Table S4). Although the KOH/C = 1.0 series exhibited higher specific surface area and pore volume at several heat-treatment temperatures, the KOH/C = 0.5 series was selected for detailed structural and electrical characterization because it provided a more favorable balance among KOH consumption, carbon yield, product purity, and mesopore development.

### 2.3. Physicochemical Characterization

X-ray diffraction (XRD) was recorded on a Rigaku SmartLab (Rigaku Corporation, Akishima, Tokyo, Japan) (Cu Kα, 40 kV/40 mA, 10–80° 2θ, 0.02° step). The interlayer spacing (d_002_) was determined from the (002) reflection using Bragg’s law, while the crystallite thickness (Lc) and in-plane crystallite size (La) were estimated from the corresponding XRD reflections using the Scherrer equation.

Raman spectra were acquired on a WITec alpha300R (WITec GmbH, UIm, Germany) (532 nm, 0.5 mW, 50× objective) and fitted with two Lorentzian peaks in the D (1350 cm^−1^) and G (1585 cm^−1^) regions. N_2_ physisorption at 77 K (Micromeritics 3Flex, Micromeritics Instrument Corporation, Norcross, GA, USA) provided BET specific surface area (SSA) and BJH mesopore volume; samples were degassed at 250 °C for 12 h. Field-emission SEM (Hitachi S-4800, Hitachi High-Tech Corporation, Tokyo, Japan) and HR-TEM (FEI Tecnai G^2^ F30, FEI Company, Hillsboro, OR, USA, 300 kV) imaged the morphology and lattice fringes. X-ray photoelectron spectroscopy (XPS, Thermo K-Alpha, Thermo Fisher Scientific, East Grinstead, UK, Al Kα) was used to quantify surface heteroatoms. Powder electrical conductivity was measured with a Mitsubishi Loresta-GX four-point probe (Mitsubishi Chemical Analytech Co., Ltd., Yamato, Kanagawa, Japan) under uniaxial pressures up to 2000 kgf.

### 2.4. Electrode Fabrication and Cell Assembly

The cathode slurry consisted of carbon (either the selected G-SMS-1800/0.5 or commercial Super P reference carbon), and PVDF in NMP. The slurry was cast on carbon paper (Toray TGP-H-060, Toray Industries, Inc., Tokyo, Japan), dried at 80 °C for 12 h in vacuum, and punched into 12 mm disks with an active-material mass loading of 0.5–0.6 mg cm^−2^. Modified CR2032 cells with a mesh window were assembled in an Ar-filled glove box (H_2_O, O_2_ < 0.5 ppm) using Li-metal foil as the anode, a Whatman GF/D separator, and 80 µL of 1 M LiTFSI in DMAc. The assembled cells were transferred to a home-built manifold, purged with dry O_2_ (99.999%) three times, and pressurized to 2.0 atm before testing.

### 2.5. Cycling Test

Galvanostatic cycling was performed on a Neware CT-4008T cycler (Neware Technology Limited, Shenzhen, China) at 25 ± 1 °C. Cells were cycled at a current density of 250 mA g^−1^ with a fixed capacity cutoff of 1000 mA h g^−1^, and a terminal voltage limit of 2.0–4.5 V vs. Li/Li^+^ was taken as the end-of-life criterion. Only two electrodes were evaluated in the present study: the selected G-SMS-1800/0.5 cathode and a commercial Super P reference cathode, assembled and tested under identical conditions. The number of cycles sustained by each electrode is reported in Section 3.5.

## 3. Results and Discussion

### 3.1. Char Formation, Yield, and the Porosity

Thermogravimetric analysis shows three mass-loss regimes for SMS: dehydration (<150 °C), hemicellulose/cellulose devolatilization (200–380 °C), and lignin condensation (400–700 °C) (Figure S1). The biochar yield after primary carbonization at 800 °C was 31.5 wt% on a dry basis and increased to 34.4 wt% under the optimized condition of 1 h with a heating rate of 3 °C min^−1^. Higher carbonization temperatures decreased the yield, while the ash content exhibited an inverted volcano-type trend, reaching its minimum at approximately 700–800 °C (Tables S2 and S3). This yield is comparable to those reported for hardwood-derived precursors (24–31 wt%) [25].

The fixed-carbon content of the char increased from 76.3 wt% at 500 °C to a maximum of 82.4 wt% at 800 °C. The O/C and H/C atomic ratios decreased monotonically with increasing carbonization temperature, holding time, and heating rate, consistent with progressive deoxygenation and aromatization. These changes are summarized in the Van Krevelen diagrams for different carbonization conditions: (a) temperature, (b) holding time, and (c) heating rate (Figure S2). On the basis of these results, carbonization at 800 °C for 1 h with a heating rate of 3 °C min^−1^ was selected as the most suitable condition.

The subsequent KOH-assisted graphitization step decreased the production yield from 58.6% at 1200 °C to 52.2% at 1800 °C, with the yield expressed relative to the carbonized char. This decrease is attributed to the well-known KOH etching reactions (6 KOH + 2 C → 2 K + 3 H_2_ + 2 K_2_CO_3_; K_2_CO_3_ → K_2_O + CO_2_ above 700 °C), which generate porosity at the expense of carbon mass [26].

The N_2_ adsorption–desorption isotherms exhibit Type IV behavior with pronounced H4 hysteresis, indicating the presence of slit-like mesopores (Figure S3). The BET specific surface area decreased from 933 m^2^ g^−1^ at 1200 °C to 880 m^2^ g^−1^ at 1400 °C, 419 m^2^ g^−1^ at 1600 °C, and 131 m^2^ g^−1^ at 1800 °C, while the mesopore fraction increased from 41.3% to 96.9% over the same temperature range (Table 1). Cumulative BJH pore-size distributions show that the modal pore diameter shifted from 2.4 nm at 1200 °C to 2.7 nm at 1400 °C and 3.8 nm at 1800 °C.

Because BJH analysis has limitations in representing the exact dimensions of slit-like pores in carbonaceous materials, the BJH-derived pore sizes and mesopore fractions are used here primarily as comparative descriptors of pore-structure evolution rather than as absolute geometrical values. The mesopore fraction was calculated as the BJH-derived mesopore volume relative to the total pore volume.

The progressive decrease in the BJH-derived micropore contribution with increasing heat-treatment temperature therefore indicates an increasing relative contribution of mesopores to the pore structure. Such mesoporosity is expected to facilitate electrolyte penetration, oxygen transport, and access to electrochemically active interfaces [6,7,27]. The characteristic mesopore size should not be interpreted as directly accommodating entire Li_2_O_2_ toroids; rather, larger discharge-product aggregates are expected to form within interparticle voids, macroporous regions, and on external carbon surfaces, while mesopores provide accessible transport pathways and reaction interfaces.

Although increasing the KOH/C ratio to 1.0 enhanced the specific surface area and pore volume, particularly at lower heat-treatment temperatures, this textural advantage became marginal at 1800 °C. The higher KOH loading also resulted in slightly larger BJH-derived characteristic pore diameters at lower treatment temperatures, consistent with enhanced carbon etching and pore widening. This difference became progressively smaller at higher temperatures, indicating that thermal restructuring increasingly contributed to pore evolution. The mesopore volumes of G-SMS-1800/0.5 and G-SMS-1800/1.0 were nearly identical at 0.31 and 0.32 cm^3^ g^−1^, respectively, whereas their corresponding production yields were 52.2% and 42.1%. Moreover, G-SMS-1800/0.5 exhibited a higher carbon content of 98.8 wt.% with ash below the detection limit, compared with 97.6 wt.% carbon and 1.7 wt.% ash for G-SMS-1800/1.0. For Li–O_2_ cathode applications, minimizing residual inorganic ash is desirable because such impurities may introduce chemically heterogeneous or electrochemically inactive surface sites and may increase the likelihood of unwanted interfacial reactions during cycling. Therefore, considering the comparable mesopore volume at 1800 °C together with the higher production yield, reduced KOH consumption, and lower residual ash, the KOH/C = 0.5 series was selected for subsequent detailed structural and electrical characterization.

### 3.2. Structural Evolution: XRD, Raman, and XPS

**Figure 1:**
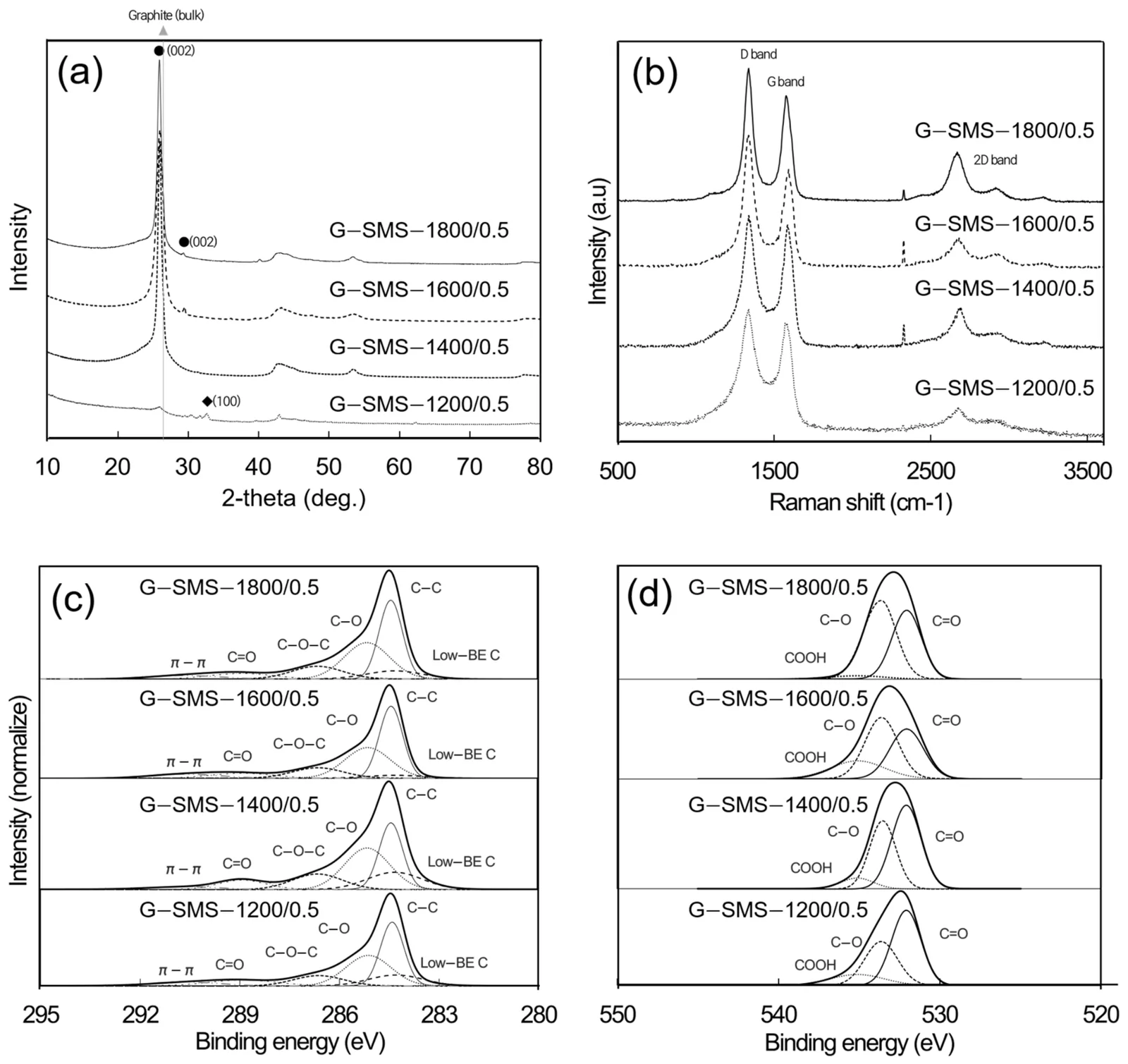
Physicochemical characterization of the G-SMS: (**a**) XRD patterns; (**b**) Raman spectra; (**c**) XPS C 1s spectra; and (**d**) XPS O 1s spectra showing the evolution with heat-treatment temperature.

The (002) reflection of C-SMS-800 is broad and centered at 2θ = 23.7°, indicative of a turbostratic structure with d_002_ = 0.390 nm. KOH-assisted heat treatment sharpens and shifts the peak toward the graphitic 26°; d_002_ decreases monotonically from 0.364 nm (1200 °C) to 0.357 nm (1400 °C) and 0.347 nm (1800 °C) (Table S5). Correspondingly, the in-plane crystallite size La derived from XRD rises from 3.6 nm to 8.2 nm. The simultaneous expansion of Lc and La together with the contraction of d_002_ demonstrates a coherent three-dimensional turbostratic-to-graphitic ordering—the graphene sheets grow both taller (more layers stacked coherently) and wider (larger defect-free basal planes)—rather than a mere loss of amorphous carbon, as schematically illustrated in Figure 1.

Raman fits give I_D/I_G ratios of 1.04 (G-SMS-1200/0.5), 0.92 (G-SMS-1400/0.5), 0.79 (G-SMS-1600/0.5) and 0.69 (G-SMS-1800/0.5). Beyond 1600 °C, a well-resolved 2D band (~2680 cm^−1^) emerges with an I_2D/I_G ratio approaching 0.5, diagnostic of a few-layer graphitic stacking sequence. A weak D + G combination band near 2940 cm^−1^ is present in all samples but decays rapidly above 1400 °C, confirming that defect-related bond disorder is progressively removed. Notably, at 1400 °C we already reach an I_D/I_G < 1—the ordered-carbon regiTme that is normally accessed only above 2000 °C without catalyst [17]—which represents a thermal-budget reduction of roughly 600–800 °C relative to non-catalytic graphitization.

To further distinguish the effect of KOH-assisted treatment from thermal annealing alone, KOH-free control samples were heat-treated under comparable conditions, and their XRD and Raman spectra were compared with those of the KOH-treated samples (Figure S5). In the absence of KOH, no clear graphite-like structural development was observed up to 1600 °C, whereas more pronounced graphitic features emerged at 1800 °C. In contrast, the KOH-treated samples exhibited noticeable structural ordering from approximately 1400 °C. These results indicate that thermal annealing itself contributes to carbon ordering, while the presence of KOH facilitates the development of graphite-like ordering at comparatively lower treatment temperatures. Therefore, the observed structural evolution is interpreted as KOH-assisted ordering rather than being attributed solely to thermal annealing [21,22].

The surface chemical structure of the carbon materials heat-treated at different temperatures was examined by X-ray photoelectron spectroscopy. The corresponding XPS survey spectra are presented in Figure S4, showing that carbon and oxygen are the predominant surface elements in all G-SMS samples, with only a minor nitrogen contribution. As the heat-treatment temperature increased, the relative oxygen signal decreased while the carbon signal became increasingly dominant, consistent with progressive deoxygenation and carbon enrichment. The high-resolution C 1s spectra further show that the sp^2^ C=C component (284.0–284.5 eV) increases progressively with heat-treatment temperature, whereas the sp^3^/disordered carbon contribution (284.5–285.0 eV) decreases.

At the same time, the surface oxygen functionalities, namely C–O (≈286.0 eV) and C=O (288–289 eV), are progressively reduced to a minimum, suppressing carbonate (Li_2_CO_3_) formation sites that act as parasitic, hard-to-decompose products during cycling. Since a higher sp^2^ fraction ensures better electronic conductivity and minimized oxygen functionalities favor a reversible, Li_2_O_2_-dominated discharge product, the sample heat-treated at G-SMS-1800/0.5, which exhibits the highest sp^2^ fraction and the lowest surface oxygen content, is identified as the most suitable cathode carbon for lithium–air batteries.

### 3.3. Morphology and Lattice Imaging

TEM shows that G-SMS-1800/0.5 preserves the hierarchical fibrous morphology of the parent lignocellulose, with 5–30 µm long carbon ribbons perforated by open macropores that provide a network of oxygen-transport channels. HR-TEM resolves 5–10 lattice fringes with a spacing of 0.347 nm curling around 3–8 nm mesopores; adjacent stacks are locally aligned but globally isotropic, giving a ‘graphitic-mosaic’ texture that combines the electronic connectivity of graphite with the accessible surface of an activated carbon. Selected-area electron diffraction shows a diffuse (002) ring superimposed on discrete (100) and (110) arcs, consistent with the coexistence of nano-graphitic domains and residual turbostratic regions.

### 3.4. Electrical Conductivity and Combined Structural Characteristics

**Figure 2:**
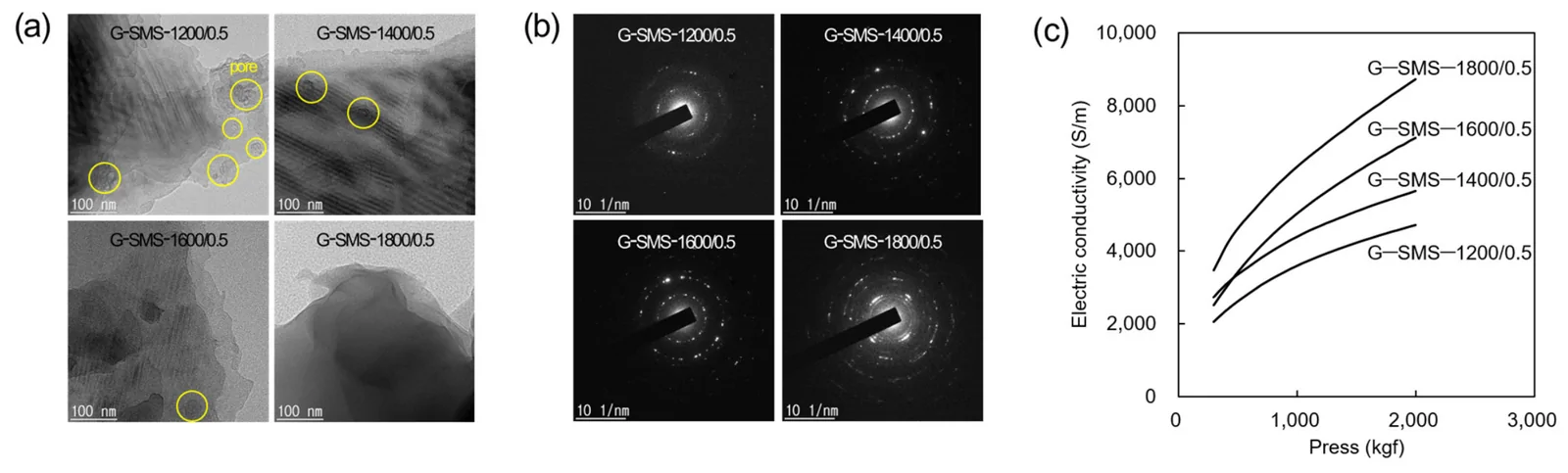
Physicochemical characterization of the G-SMS heat-treated at different temperatures: (**a**) SEM images; (**b**) HR-TEM; (**c**) electrical conductivity.

Powder conductivity rises monotonically along the KOH/C = 0.5 series from 4313 S m^−1^ (1200 °C) to 6982 S m^−1^ (1400 °C), 8120 S m^−1^ (1600 °C), and 8607 S m^−1^ (1800 °C) (Table 2 and Figure 2c). To provide a simple comparison of the concurrent evolution of mesopore volume and electrical conductivity, the product V_Meso × σ was additionally calculated. This value increases monotonically across the series—819 (1200 °C), 1536 (1400 °C), 2274 (1600 °C), and 2668 cm^3^ g^−1^ S m^−1^ (1800 °C)—and reaches its highest value for G-SMS-1800/0.5 (Table 2). Because both V_Meso and σ increase with heat-treatment temperature over the investigated range, this product is used here only as a comparative index and not as an independent criterion for identifying an optimum cathode condition.

G-SMS-1800/0.5 also combines the smallest interlayer spacing (d_002_ = 0.347 nm), the lowest I_D/I_G ratio (0.69), the largest mesopore fraction (96.9%), the highest purity (carbon content 98.8 wt.%, with ash below the detection limit; Table S4), and the largest in-plane crystallite size (Table S5). Thus, the selection of G-SMS-1800/0.5 for cell-level evaluation was based on the overall convergence of these independently measured structural and electrical characteristics within the investigated temperature range, rather than on V_Meso × σ alone. G-SMS-1800/0.5 was accordingly selected as the representative SMS-derived carbon for subsequent cell-level evaluation.

### 3.5. Cycling Stability Versus Commercial Super P

Based on the structural and electrical characteristics discussed above, G-SMS-1800/0.5 was selected as the representative SMS-derived carbon for cell-level evaluation and was directly compared with commercial Super P under identical cycling conditions. No other SMS-derived sample was evaluated electrochemically. One independently assembled cell was tested for each cathode material (n = 1), and the resulting cycle numbers therefore represent a direct comparative assessment rather than a statistical evaluation of cycling reproducibility.

**Figure 3:**
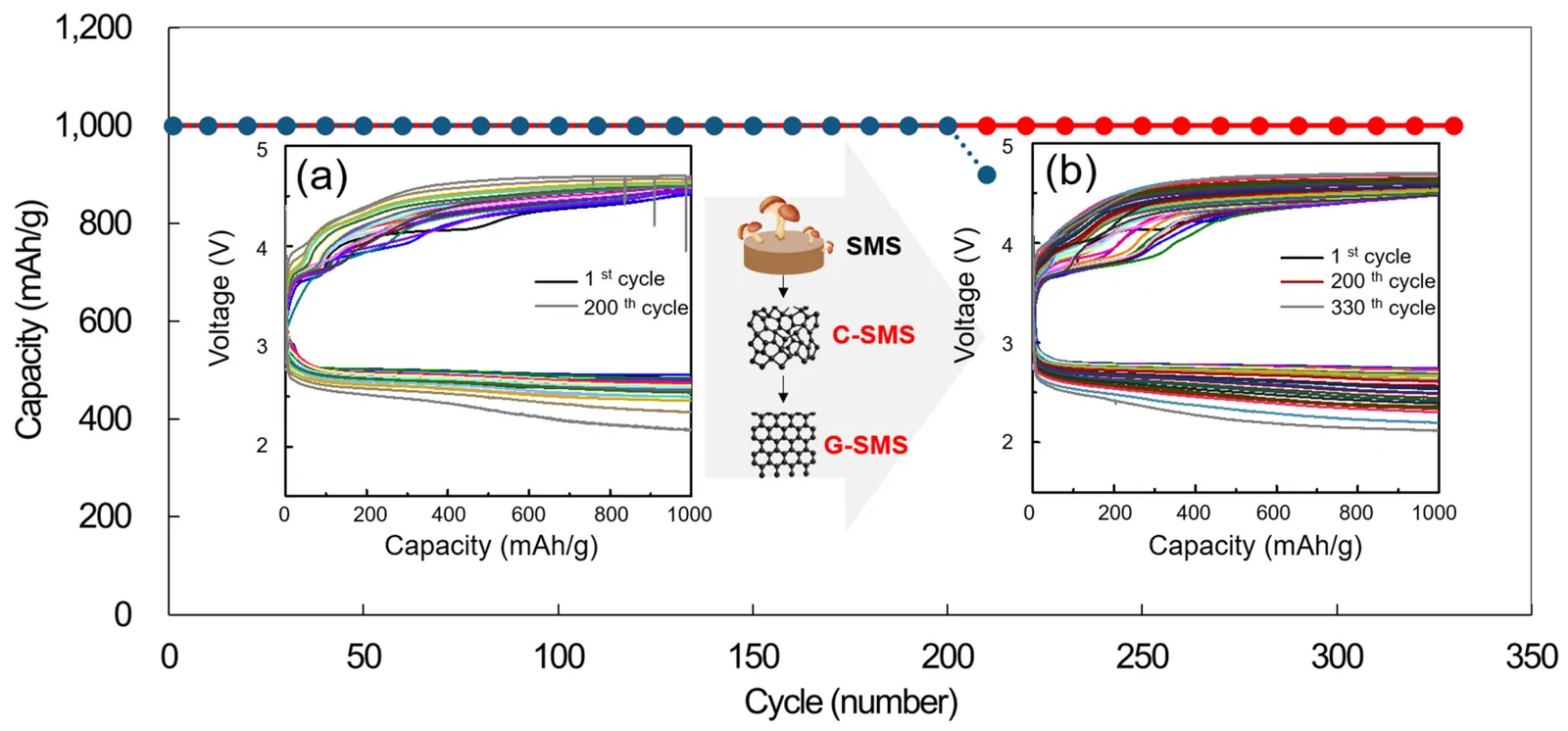
Galvanostatic discharge/charge cycling results of the Li–O_2_ batteries with a capacity/voltage cut-off condition ([250 mA g^−1^, 1000 mA h g^−1^ cut-off, voltage 2.0–4.5 V]): (**a**) Super P cathode and (**b**) G-SMS-1800/0.5 cathode.

Under the fixed-capacity cycling protocol, G-SMS-1800/0.5 completed 330 cycles, whereas the Super P reference cathode completed 200 cycles, corresponding to an approximately 1.65-fold difference in the number of completed cycles (Figure 3). In addition to cycle number, the evolution of the charge–discharge voltage gap was used to assess the development of polarization during cycling. At the first cycle, both cathodes exhibited similar charge and discharge voltages of approximately 4.5 and 2.7 V, respectively, corresponding to a voltage gap of approximately 1.8 V. By the 200th cycle, the voltage gap of Super P increased to approximately 2.5 V, with charge and discharge voltages of approximately 4.7 and 2.2 V, respectively. In contrast, G-SMS-1800/0.5 exhibited charge and discharge voltages of approximately 4.6 and 2.6 V at the 200th cycle, corresponding to a smaller voltage gap of approximately 2.0 V. The smaller increase in voltage separation for G-SMS-1800/0.5 indicates lower polarization growth and better preservation of charge/discharge reversibility during prolonged cycling. The origin of this difference may be related to the structural characteristics summarized in Table 2. G-SMS-1800/0.5 exhibits a relatively low I_D/I_G ratio (0.69), a small d_002_ value (0.347 nm), and a mesopore-dominated structure with a mesopore fraction of 96.9% and a mesopore volume of 0.31 cm^3^ g^−1^. These characteristics are consistent with the lower polarization growth observed during prolonged cycling. However, because the present electrochemical comparison was limited to G-SMS-1800/0.5 and Super P under identical test conditions, the contribution of individual structural parameters to cycling durability cannot be independently distinguished from the present results.

### 3.6. Sustainability and Scope of the Present Study

A comparison with representative biomass-derived Li–O_2_ cathodes is summarized in Table 3. Because the reported studies employ different electrolytes, current densities, capacity cutoffs, oxygen conditions, and cell configurations, the comparison is intended to highlight differences in material-design strategies rather than to provide a direct numerical ranking of cycling performance. Within this context, G-SMS-1800/0.5 is distinguished by the combination of graphite-like ordering, a mesopore-dominated carbon framework, and high electrical conductivity achieved without heteroatom doping, transition-metal co-catalysts, templates, post-synthetic functionalization. The observed 330-cycle performance therefore demonstrates the potential of an undoped, metal-free, and template-free biomass-derived carbon cathode, although direct quantitative comparison with literature values should be interpreted cautiously.

The proposed process may reduce some of the thermal and material requirements associated with conventional synthetic graphite production. The peak treatment temperature of 1800 °C is substantially lower than that of industrial Acheson graphitization (~2800 °C), and an interlayer spacing of 0.347 nm was achieved at 1800 °C, whereas non-catalytic graphitization typically requires ≥2500 °C [17]. The thermal-energy demand of synthetic-graphite production has been well documented [8,9]. In addition, SMS represents an abundant agro-industrial waste feedstock, and the present two-step protocol uses KOH, N_2_, and dilute HCl without organic solvents, templates, or transition-metal catalysts, suggesting potential compatibility with existing carbonization infrastructure. A dedicated cradle-to-gate life-cycle assessment and pilot-scale techno-economic analysis would be required to quantitatively assess the environmental and economic advantages of the process.

Several limitations define the scope of this study. First, the cycling test was conducted under pure O_2_ at 2.0 atm, and therefore performance under ambient air containing H_2_O and CO_2_ remains to be established. Second, the LiTFSI/DMAc electrolyte is convenient for benchmarking but is not fully stable against reduced-oxygen intermediates during extended cycling. Third, post-cycling identification of the discharge and charge products was not performed; therefore, suppression of parasitic reactions and reversible Li_2_O_2_ formation were not directly demonstrated in the present study. Finally, batch-to-batch variability in SMS composition may influence the resulting carbon microstructure and should be examined across commercially relevant SMS sources before industrial application.

## 4. Conclusions

A single-reagent, KOH-assisted route converted spent mushroom substrate, a high-volume agro-industrial waste, into a graphite-like porous carbon suitable as a lithium–oxygen battery cathode. Following carbonization of the SMS derived from Pleurotus ostreatus at 800 °C, a KOH/C mass ratio of 0.5 was selected as a practical compromise between reagent consumption, carbon yield, purity, and textural properties. From 1200 to 1800 °C the crystallinity improved monotonically: d_002_ contracted from 0.364 to 0.347 nm, I_D/I_G fell from 1.04 to 0.69, the mesopore fraction rose from 41.3% to 96.9%, and the powder conductivity increased from 4313 to 8607 S m^−1^. The mesopore-volume-weighted conductivity descriptor V_Meso × σ reached its maximum (2668 cm^3^ g^−1^ S m^−1^) for G-SMS-1800/0.5, identifying this sample as the structurally most robust host of the series. In a direct cell-level comparison under identical conditions, G-SMS-1800/0.5 sustained 330 cycles whereas commercial Super P sustained only 200. KOH-assisted heat treatment promoted graphite-like structural ordering, achieving an interlayer spacing of 0.347 nm at 1800 °C, while simultaneously generating a mesopore-dominated carbon framework. This combined structural evolution highlights the potential of KOH-assisted processing to reduce the thermal requirement for biomass graphitization without invoking a specific potassium-intercalation mechanism. The relatively ordered carbon structure and mesopore-dominated texture of G-SMS-1800/0.5 are consistent with its improved cycling behavior compared with Super P under the present test conditions, demonstrating the potential of spent mushroom substrate as a waste-derived precursor for metal–air cathode carbons.

The present study focuses on material performance at the laboratory scale; therefore, the environmental and economic feasibility of the process, including chemical consumption, washing effluent, drying, and high-temperature operation, requires further evaluation through dedicated LCA and TEA.

## Figures and Tables

**Table 1:** Textural properties for samples at different graphitization conditions.

KOH/C = 0.5	G-SMS-1200/0.5	G-SMS-1400/0.5	G-SMS-1600/0.5	G-SMS-1800/0.5
S_BET_ (m^2^/g)	933	880	419	131
V_Total_ (cm^3^/g)	0.46	0.44	0.33	0.32
V_Meso_ (cm^3^/g)	0.19	0.22	0.28	0.31
F_Micro_ (%)	58.7	46.3	16.9	3.13
Pore size (nm)	2.4	2.7	3.4	3.8
KOH/C = 1.0	G-SMS-1200/1.0	G-SMS-1400/1.0	G-SMS-1600/1.0	G-SMS-1800/1.0
S_BET_ (m^2^/g)	1319	1104	452	307
V_Total_ (cm^3^/g)	0.74	0.63	0.39	0.33
V_Meso_ (cm^3^/g)	0.49	0.48	0.35	0.32
F_Micro_ (%)	33.8	24.0	10.2	3.03
Pore size (nm)	2.7	3.0	3.4	3.9

**Table 2:** Key properties of G-SMS.

Sample	d_002_ (nm)	I_D/I_G	V_Meso (cm^3^ g^−1^)	σ (S m^−1^)	V_Meso × σ (cm^3^ g^−1^ S m^−1^)
C-SMS-800	0.390	1.31	0.05	510	25.1
G-SMS-1200/0.5	0.364	1.04	0.19	4313	819
G-SMS-1400/0.5	0.357	0.92	0.22	6982	1536
G-SMS-1600/0.5	0.351	0.79	0.28	8120	2274
G-SMS-1800/0.5	0.347	0.69	0.31	8607	2668

**Table 3:** Comparison of representative biomass-derived graphitized carbons and graphitic carbon cathodes relevant to Li–O_2_ batteries.

Precursor	Processing/Modification	Max. T (℃)	BET SSA (m^2^/g)	Electrochemical Conditions	Cycling Performance	Ref.
Coconut coir waste	KOH/Ni assisted catalytic graphitization	1200	162.31	LIB anode	397.6 mAh g^−1^,	[28]
Biomass precursors	High temperature carbonization/graphitization	2800	-	LIB anode	Improved Li-storage with graphitization	[25]
Bamboo charcoal	Direct high temperature carbonization/graphitization	2400	8.45	LIB electrode; 0.02 A g^−1^	217 mAh g^−1^ reported for highly graphitized charcoal	[29]
Graphene-based carbon	Template synthesis + high temperature annealing	1800	-	Li-O_2_ cathode	>6700 mAh g^−1^ capacity; improved cycling attributed to edge-site-free/topological-defect-rich framework	[30]
Spent mushroom substrate (SMS)	KOH-assisted graphitization	1800	131 96.9% mesopore	Li-O_2_ cathode	330 cycles	This work

## Data Availability

The original contributions presented in this study are included in the article/Supplementary Material. Further inquiries can be directed to the corresponding author.

## Supplementary Materials

The following supporting information can be downloaded at https://www.mdpi.com/article/10.3390/ma19214632/s1: Table S1: The results of proximate analysis and ultimate analysis of spent mushroom substrate (SMS); Table S2: Proximate and ultimate analyses of C-SMS at various carbonization temperatures; Table S3: Proximate and ultimate analyses of C-SMS at various carbonization holding time and ramping rate; Figure S1: Thermogravimetric analysis shows three mass-loss regimes for SMS; Figure S2: The O/C and H/C atomic ratios of C-SMS are presented in a Van Krevelen diagram with different carbonization conditions (a) temperature, (b) holding time, and (c) ramping rate; Table S4: Proximate and ultimate analyses of the G-SMS series; Figure S3: N_2_ adsorption–desorption isotherms showing hysteresis loops of the G-SMS with different temperatures with activator ratios: (a) K/C = 0.5, (b) K/C = 1.0; Figure S4: XPS survey spectra of G-GMS-K/C 0.5; Figure S5: Comparison of the structural evolution of SMS-derived carbons without KOH treatment at different heat-treatment temperatures: (a) XRD patterns and (b) Raman spectra; Table S5. Structural parameters of G-SMS-derived graphite-like materials.
