# Supplementary material for: Biomass-derived Graphite-like Carbon as a Sustainable Cathode Material for Lithium Oxygen Batteries
Source: Materials (Basel). 2026 Oct 31;19(21):4632. doi: 10.3390/ma19214632 (PMC13765017; doi:10.3390/ma19214632)
Supplement: Supplementary file 1 [file materials-19-04632-s001.zip › materials-4659367-supplementary.pdf]

Biomass-derived Graphite-like Carbon as a Sustainable Cathode Material for Lithium Oxygen Batteries

Da-Jung Kang[1], Cheol-Hui Ryu[2], Gwang-Hee Lee[2], Kwang-Mo Seong[3], and Jung-Eun Park[2],†

[1]Center of Bio-resources Recycling, Institute for Advanced Engineering, Yongin 17180, Republic of Korea

[2]Center of Materials Science and Chemical Engineering, Institute for Advanced Engineering, Yongin 17180, Republic of Korea

[3] Interior Materials Development Team, Hyundai Motor Group, 150, Hyundaiyeonguso-ro, Namyang-eup, Manse-gu, Hwaseong-si 18280, Republic of Korea

†Corresponding author: jepark@iae.re.kr

Supplementary Materials

Table S1. The results of proximate analysis and ultimate analysis of spent mushroom substrate (SMS).

Composition	Proximate analysis			Ultimate analysis			
	Volatile	Fixed carbon	Ash	Carbon	Hydrogen	Nitrogen	Oxygen
(%)	79.6	18.0	2.4	47.6	5.8	1.2	42.9

Table S2. Proximate and ultimate analyses of C-SMS at various carbonization temperatures.

Temperature (°C)		SMS	500	600	700	800	900	1000
*Biochar yield (%)		-	43.3	33.0	31.8	31.5	31.3	29.5
Proximate analysis (%)	Volatile	79.6	13.7	9.7	9.0	7.4	4.9	4.6
	Fixed carbon	18.0	76.3	79.6	80.8	82.4	75.6	79.4
	Ash	2.4	9.9	10.8	10.2	10.3	19.6	16.1
Ultimate analysis (%)	Carbon	47.6	76.2	79.1	79.1	80.2	77.6	78.7
	Hydrogen	5.8	2.8	1.9	1.1	0.7	0.9	0.3
	Oxygen	42.9	8.9	6.5	8.7	6.5	0.2	3.9
	Nitrogen	1.2	2.0	1.7	0.8	1.7	2.0	1.9
	Sulfur	0.0	0.0	0.0	0.0	0.0	0.0	0.0

Table S3. Proximate and ultimate analyses of C-SMS at various carbonization holding time and ramping rate.

Temperature (°C)		800	800	800	800	800	800
Holding time (hr)		1	1	1	1	3	5
Ramping rate (°C/min)		1.5	3.0	5.0	10.0	5.0	5.0
*Biochar yield (%)		33.0	34.4	30.6	31.0	31.5	29.1
Proximate analysis	Volatile	10.0	10.4	14.8	15.9	6.4	5.8
	Fixed carbon	80.1	80.1	79.7	76.1	82.4	80.6
	Ash	9.9	9.5	9.5	8.0	11.3	13.5
Ultimate analysis	Carbon	77.0	81.5	77.2	79.6	80.2	81.7
	Hydrogen	1.0	0.8	0.9	0.9	0.7	0.8
	Oxygen	10.6	7.3	6.2	10.5	6.5	3.0
	Nitrogen	1.5	1.0	1.3	1.0	1.7	1.0
	Sulfur	0.0	0.0	0.0	0.0	0.0	0.0

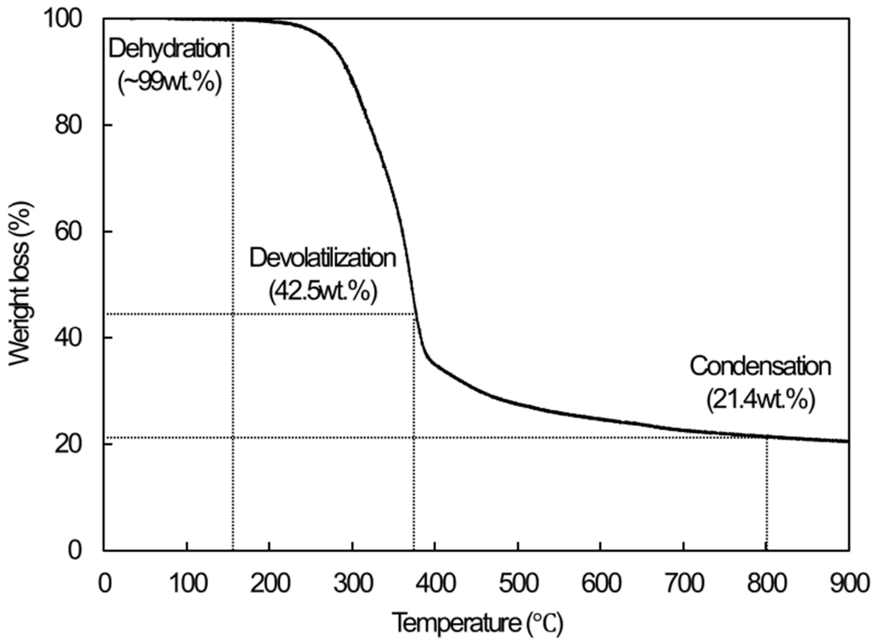

Figure S1. Thermogravimetric analysis shows three mass-loss regimes for SMS

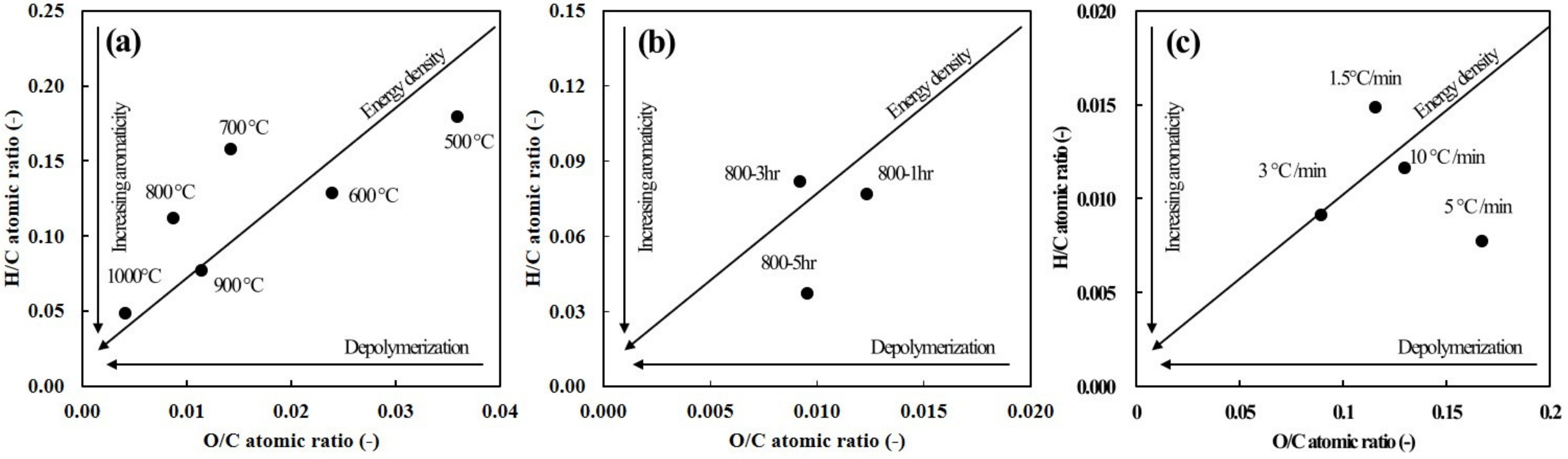

Figure S2. The O/C and H/C atomic ratios of C-SMS are presented in a Van Krevelen diagram with different carbonization conditions (a) temperature, (b) holding time, and (c) ramping rate.

Table S4. Proximate and ultimate analyses of the G-SMS series.

KOH/C=0.5	G-SMS-1200/0.5	G-SMS-1400/0.5	G-SMS-1600/0.5	G-SMS-1800/0.5
Production yield (%)	58.6	57.7	54.1	52.2
Fixed Carbon (wt.%)	77.7	86.3	88.2	94.4
Volatile (wt.%)	11.5	9.4	9.4	5.6
Ash (wt.%)	10.8	4.3	2.4	0.0
Carbon (wt.%)	81.3	91.9	96.0	98.8
Hydrogen (wt.%)	0.2	0.1	0.1	0.1
Oxygen (wt.%)	7.2	3.2	1.0	0.7
Nitrogen (wt.%)	0.5	0.5	0.5	0.4
Sulfur (wt.%)	0	0	0	0
KOH/C=1.0	G-SMS-1200/1.0	G-SMS-1400/1.0	G-SMS-1600/1.0	G-SMS-1800/1.0
Production yield (%)	47.3	47.0	43.1	42.1
Fixed Carbon (wt.%)	73.3	82.0	85.7	90.9
Volatile (wt.%)	12.1	12.3	10.9	7.4
Ash (wt.%)	14.6	5.7	3.4	1.7
Carbon (wt.%)	81.0	92.5	95.1	97.6
Hydrogen (wt.%)	0.2	0.1	0.1	0.0
Oxygen (wt.%)	3.7	1.2	1.0	0.3
Nitrogen (wt.%)	0.5	0.5	0.4	0.4
Sulfur (wt.%)	0.0	0.0	0.0	0.0

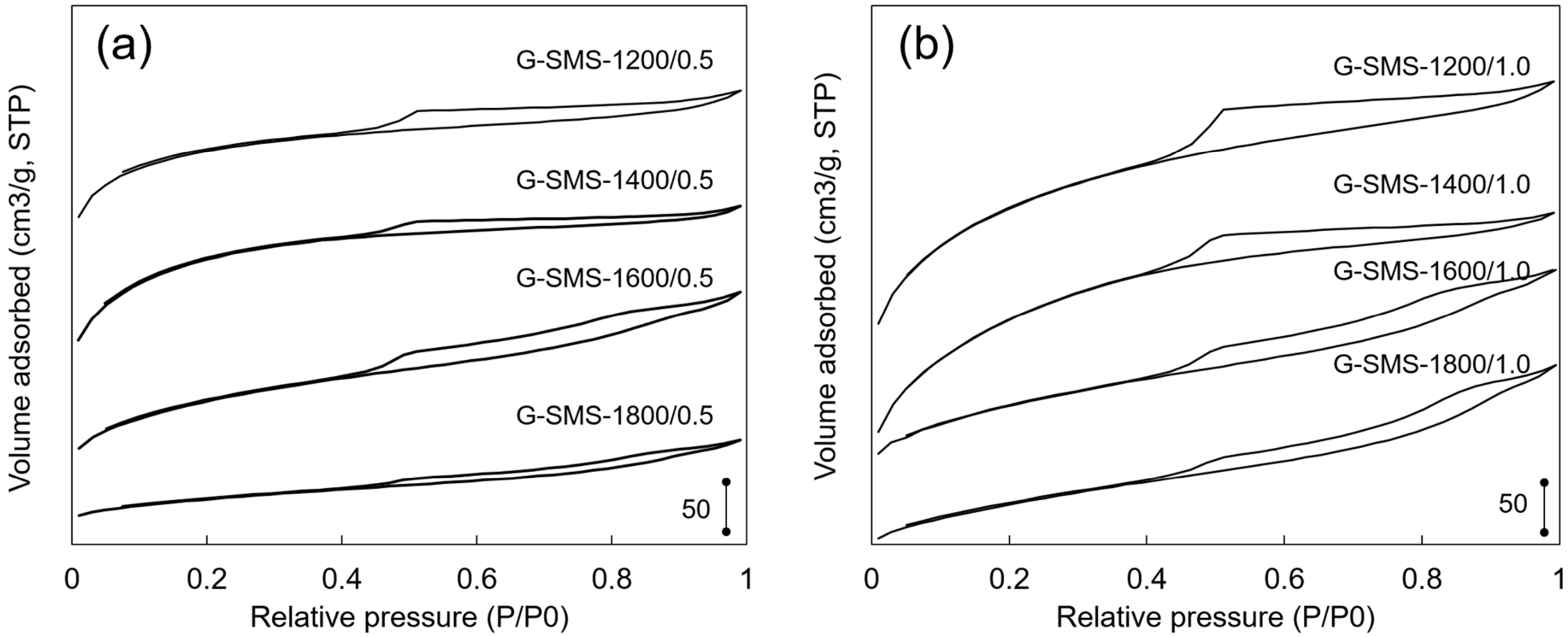

Figure S3. N_2 adsorption–desorption isotherms showing hysteresis loops of the G-SMS with different temperatures with activator ratios: (a) K/C = 0.5, (b) K/C = 1.0.

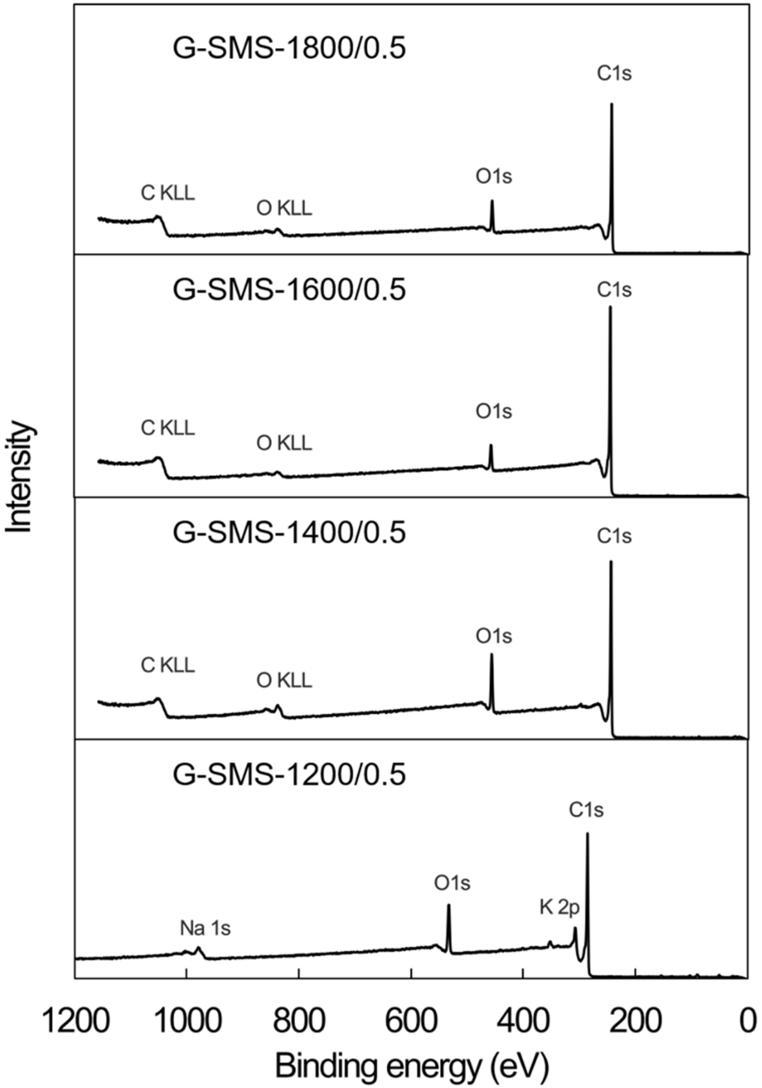

Figure S4. XPS survey spectra of G-GMS-K/C 0.5.

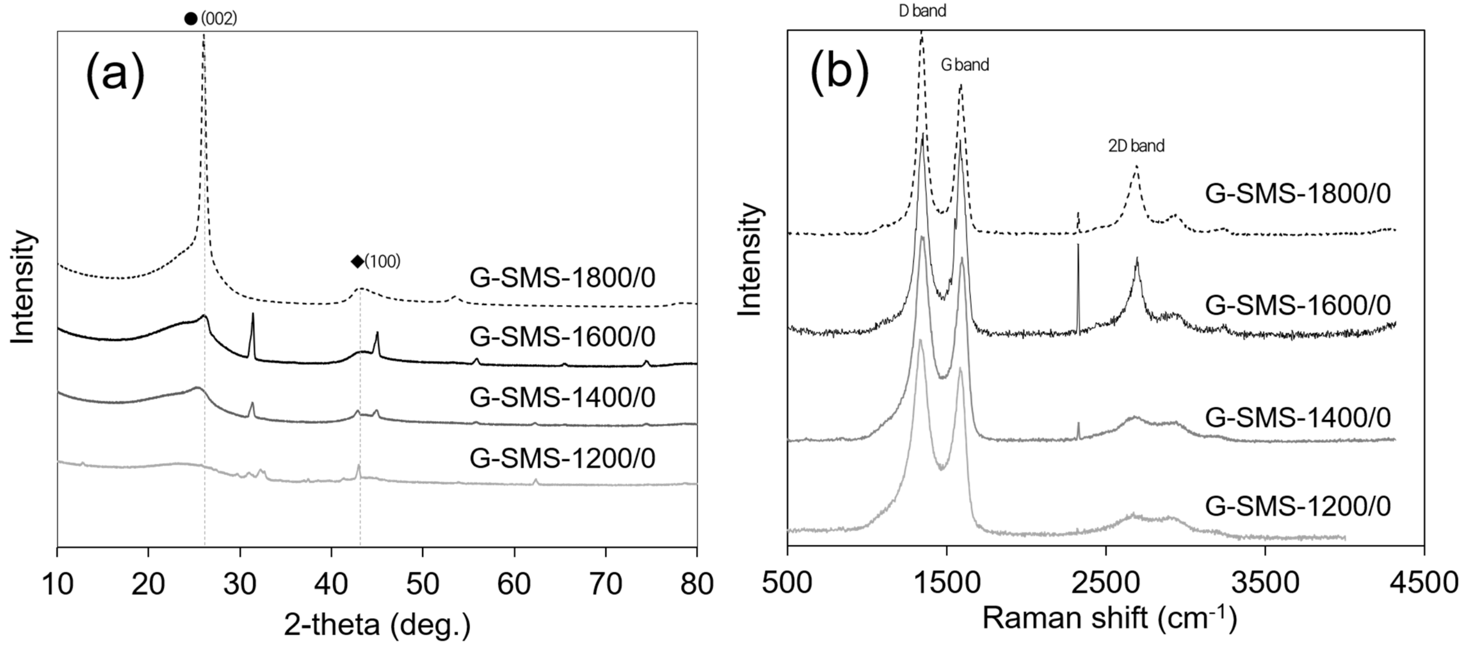

Figure S5. Comparison of the structural evolution of SMS-derived carbons without KOH treatment at different heat-treatment temperatures: (a) XRD patterns and (b) Raman spectra.

Table S5. Structural parameters of G-SMS-derived graphite-like materials.

Sample	XRD			RAMAN			XPS		
	d_{002} (nm)	L_c (nm)	L_a (nm)	I_D	I_G	I_D/I_G	O1s	C1s	N1s
G-SMS-1200/0.5	0.364	1.56	3.61	52.24	50.04	1.04	15.22	84.03	0.76
G-SMS-1400/0.5	0.357	1.58	4.31	51.01	55.20	0.92	13.91	85.76	0.33
G-SMS-1600/0.5	0.351	2.27	4.49	47.84	60.07	0.79	9.62	89.82	0.57
G-SMS-1800/0.5	0.347	2.98	8.17	32.49	47.00	0.69	6.82	92.75	0.43
